# Complex health-related needs among young, soon-to-be-released prisoners

**DOI:** 10.1186/2194-7899-1-1

**Published:** 2013-10-24

**Authors:** Kate van Dooren, Alun Richards, Nick Lennox, Stuart A Kinner

**Affiliations:** 1 Queensland Centre for Intellectual and Developmental Disability, School of Medicine, The University of Queensland, Brisbane, Australia; 2 Blood Borne Viruses and Sexually Transmissible Infections, Queensland Department of Health, Brisbane, Australia; 3 Centre for Health Policy, Programs and Economics, Melbourne School of Population Health The University of Melbourne, Melbourne, Australia; 4 School of Medicine, The University of Queensland, Brisbane, Australia; 5 School of Public Health and Preventive Medicine, Monash University, Melbourne, Australia; 6 Murdoch Children’s Research Institute, Melbourne, Australia

**Keywords:** Prisoners, Young adult, Risk-taking, Morbidity, Illicit drugs

## Abstract

**Background:**

To estimate the prevalence and co-occurrence of health-related needs among young people aged 18 to 24 years transitioning out of adult prisons.

**Methods:**

Data came from face-to-face, confidential interviews with adult prisoners aged 18–24 years in seven adult prisons in Queensland, Australia. We identified the prevalence and co-occurrence of overlapping health-related needs using an Australian health performance framework with four domains: physical health, mental health, risky substance use and socioeconomic disadvantage.

**Results:**

Most young prisoners experience multiple and complex health problems prior to their release: 98% of young prisoners reported at least one indicator of poor health, and 30% reported at least one indicator of poor health in all four evaluated domains.

**Conclusions:**

Young people in adult prisons report a high prevalence of health problems across multiple domains. Addressing these complex needs will require coordinated service delivery across health-related sectors both in custody and after release.

## Background

The transition out of prison is challenging, with most individuals re-entering the community with entrenched and overlapping health and social needs (Hammett et al. 2001; Kinner 2006; Kinner and Cogger 2007; Mallik-Kane and Visher 2008). Post-release, risk of death from drug overdose and suicide is high and morbidity is greatly elevated compared with the general population (Bird and Hutchinson 2003; Kariminia et al. 2007; Merrell et al. 2010). A substantial proportional of individuals reoffend and are reincarcerated within a relatively short period (Gendreau et al. 1996; Baldry et al. 2004; Holland et al. 2007; Wilson and Zozula 2012). Many of the individual and structural factors that influence post-release health-related outcomes are also relevant from a criminal justice perspective: those who experience homelessness, unemployment, alcohol and drug misuse and lack of social connectedness are likely to experience poor health outcomes and are also more likely to re-offend (Borzycki and Baldry 2003; Baldry et al. 2004). Therefore, interventions that successfully target these factors during and after the transition from custody have the potential to simultaneously improve health outcomes and reduce recidivism. A prerequisite for the development of such interventions is a detailed understanding of the unaddressed health needs of soon-to-be-released prisoners (Ahalt et al. 2012).

In response to long-standing gaps in knowledge about the health of prisoners, Australia’s national agency for health and welfare statistics established a set of national prisoner health indicators (NPHI) that are now collected in prisons nationally and reported annually (Belcher and Al Yaman 2007; Australian Institute of Health and Welfare 2011a). The NPHI are used to inform service delivery in prisons and provide a benchmark for measuring changes in health status and service delivery over time. However, from a re-entry perspective, the NPHI are limited in two important ways. First, the focus of the NPHI is prisoner health and prison health services, based primarily on a census of prison receptions, such that the collection is likely to be a poor reflection of the health status of *soon-to-be released* prisoners. Second, reports based on the NPHI consider each health indicator separately, failing to identify or characterise overlapping health needs. Consequently, we remain ill-informed about the co-occurrence or concentration of health needs among soon-to-be-released prisoners and lack data to inform co-ordinated re-entry programs.

In the community, Australians have access to two national health care systems: Medicare, which affords subsidised treatment from medical practitioners, including general practitioners and allied health professionals; and the Pharmaceutical Benefits Scheme, which subsidises certain prescribed pharmaceuticals. However, correctional health care is not a federal legislative responsibility; instead responsibility for correctional health care falls to each state and territory and prisoners lose access to these universal health care systems on entry into prison, which are then reinstated on release (Australian Institute of Health and Welfare 2011a Kinner et al. 2012). Although prison provides the opportunity to access medical treatment to a group who typically underuse health services in the community, these exclusions preclude financial incentive for community-based health services providers to enter prisons and act as a barrier to continuity of care during the transition out of prison (Kinner et al. 2012).

Further, implicit in some correctional policies and programs is the view that prisoners are a relatively homogeneous group, defined by their shared experiences of incarceration (van Dooren et al. 2011). However, intervention studies with incarcerated populations typically find important subgroup differences in both treatment needs and treatment outcomes (Lattimore and Visher 2009; Friedmann et al. 2012; Wilson and Zozula 2012). Recently, young people aged 18–24 years have been identified as a subgroup among adult prisoners who might have different health-related (and consequently, health service) needs, because their developmental needs and sociocultural experiences differ substantially from those of older adults (Farrington et al. 2012; Cauffmann 2012; Woolard 2012). Little is known about the re-entry experiences of this group, although some authors have speculated that they might need more support to deal with drug and alcohol, mental health and employment issues than their older peers (van Dooren et al. 2013). The imperative for young people to mature into active and healthy adults and exit ‘the revolving door of prison’ makes their health experiences an important concern.

To better inform re-entry programming for young people, in this study we describe the prevalence and co-occurrence of health-related needs in a large sample of young, soon-to-be released prisoners. To permit comparison with national data, we restrict our analysis to health-related variables included in Australia’s NPHI framework.

## Methods

This study uses data from face-to-face, confidential interviews that took place between August 2008 and July 2010 with adult prisoners aged 18–24 years in seven adult prisons in Queensland, Australia. Trained researchers, independent of Queensland Corrective Services conducted all interviews, which provided baseline data for a randomized controlled trial of an intervention designed to improve health outcomes for ex-prisoners (Kinner 2008; Kinner et al. 2009). Randomization occurred after baseline data were collected. For the purposes of this paper, eligible participants were sentenced prisoners aged <25 years who were within six weeks of expected release from custody at the time of interview, and who provided informed, written consent to participate. Consistent with Australia’s NPHI sampling frame (Australian Institute of Health and Welfare [Bibr CR5][Bibr CR43]), our exclusion criteria was limited to on remand (pre-trial detainees) and having participated previously in the trial. No potential participant was excluded due to inability to provide informed consent and very few were judged unsafe to approach for interview. Among eligible participants, the main reasons for non-participation were lack of interest in participating and a desire not to be contacted after release from custody. Females were oversampled to improve statistical power for sex-specific analyses.

The data collection tool included 10 sections including demographic and criminographic characteristics and pre-incarceration living circumstances; physical health, mental health and health-related quality of life; and patterns of alcohol, tobacco and other drug use and risky substance use behaviors. Interviews typically took 60–90 minutes to complete. Ethical clearance for the study was granted by The University of Queensland’s Behavioural and Social Sciences Ethical Review Committee.

### Mapping morbidity

We based our analyses on the NHPI, which was created to better understand prisoner health across different points of incarceration and maps morbidity across three levels. Within each level are domains containing measurable indicators of prisoner health. *Level 1* represents the health status of prisoners (e.g., *domain*: physical health conditions, *indicator*: hepatitis C virus infection); *Level 2* represents factors influencing health and wellbeing (e.g., *domain*: socioeconomic factors, *indicator*: educational attainment); and *Level 3* represents performance of the health system (e.g., *domain*: continuity of care, *indicator*: health-related discharge planning). We limited our analyses to the first two levels; overlapping morbidity across these levels can complicate treatment in prison and in the community (Borzycki and Baldry 2003; Fazel et al. 2006) due to the need for coordinated provision of services from multiple, overlapping sectors (Hammett et al. 2001).

Consistent with the NHPI, we mapped selected variables from the survey against four domains of health need, including two health status domains: physical health and mental health; and two health risk factor domains: socioeconomic disadvantage and risky substance use behaviors. We limited indicators in each domain to those identified in the NHPI, unless explicitly stated below. In this study, we report a mix of current, pre-incarceration and lifetime indicators. Where we chose lifetime health indicators, instead of more recent indicators (for example one or three month previously) we did so because we collected data from prisoners at the end of their sentence, when they were most likely to have had their health issues addressed. Given evidence that historical health problems in prisoners often recur soon after release (Kinner 2006; Peterman et al. 2006) we believe that ‘current’ variables would under-ascertain health needs in this population. Furthermore, although we acknowledge that lifetime prevalence is not the same as point prevalence, for at least some of the health conditions we examine, past morbidity is a strong predictor of future morbidity. For example, past STIs are strong predictors of reinfection (Peterman et al. 2006).

### Health status domain

#### Physical health indicators

Indicators of poor physical health included: (1) lifetime history of being diagnosed with a sexually transmissible infection (STI): gonorrhea, chlamydia or genital herpes; (2) lifetime history of chronic conditions: head injuries, cancer, diabetes and cardiovascular disease; and (3) hepatitis C (HCV) infection. All participants were asked to self-report HCV status (indicating exposure to the virus), and for a subset of participants, HCV antibody test results were available from prison medical records (indicating HCV antibody status). A combined variable indicating HCV exposure by either self-report or pathology was used in all analyses.

#### Mental health indicators

Indicators of poor mental health included: (1) self-reported lifetime history of being diagnosed with a mental illness; (2) history of attempted suicide or self-harming behavior; and (3) currently reporting high or very high levels of psychological distress according to scores on the Kessler Psychological Distress Scale (K10) (Kessler et al. 2005). The K10 is widely used in population studies in Australia and internationally, due to its excellent psychometric properties (Kessler et al., 2003). This instrument is used nationally as a screener for prison receptions (Australian Institute of Health and Welfare [Bibr CR5][Bibr CR43] Australian Institute of Health and Welfare 2010).

### Health risk factor domain

#### Risky substance use indicators

Indicators of risky substance use included (1) lifetime history of injecting drug use (IDU); (2) using three or more illicit drugs in the year prior to incarceration; and (3) high risk/dependent alcohol use in the year prior to incarceration, according to scores on the Alcohol Use Disorders Identification Test (AUDIT) (Saunders et al. 1993; Babor et al. 2001).

#### Socioeconomic indicators

For socioeconomic disadvantage, the NHPI identifies only one outcome: ‘10 years of education’. Pre-incarceration accommodation status and employment status are strongly associated with re-entry ‘success’ (Gendreau et al. 1996; Baldry et al. 2004) and are important determinants of health (Australian Institute of Health and Welfare 2010 Australian Institute of Health and Welfare [Bibr CR5][Bibr CR43]), so for this domain, we selected three indicators of socioeconomic disadvantage: (1) fewer than 10 years of education, (2) unstable accommodation in the six months prior to incarceration, and (3) unemployment in the six months prior to incarceration.

### Statistical analyses

All indicators were dichotomized such that each participant either did or did not exhibit morbidity according to the indicator. Morbidity in a domain was also dichotomized, with those exhibiting morbidity according to at least one indicator in that domain classified as exhibiting morbidity in that domain.

Descriptive statistics were used to determine the prevalence of morbidity according to each indicator within each domain as well as overlapping morbidity across the different indicators and domains. All analyses accounted for the oversampling of females in the parent trial and were performed using Stata version 11 (StataCorp 2009).

## Results

The sample consisted of 376 participants with valid responses on all variables of interest. Most participants were non-Indigenous (74.9%) males (88.6%) with a history of previous adult incarceration (63.6%).

### Morbidity

#### Prevalence of morbidity

Descriptive statistics for the sample are presented in Table 1. More than one in five participants (21.7%) was HCV exposed and 40.4% reported a history of being diagnosed with a mental illness. Nearly half (44.6%) were unemployed in the six months prior to incarceration, 50.1% reported high risk/dependent patterns of alcohol use and 52.4% reported a history of IDU.

**Table 1:** **Demographic and offending characteristics, health morbidity and health risk factors**

	N = 376 n (%)
**Demographic and offending characteristics**	
Median age in years (range)	22.2 (18.0-24.9)
Female	11.4
Indigenous	25.9
Mean number juvenile incarcerations (range)	1.5 (0–33)
Mean total adult incarcerations (range)	2.9 (1–17)
**Any health morbidity**	**76.7**
*One or more marker of physical health*	*72.6*
Hepatitis C exposed	21.7
Sexually transmissable infections	16.2
Chronic conditions	8.7
*One or more marker of mental health*	*55.4*
Diagnosed mental illness	40.4
Self-harm/suicide attempt	26.7
K10 high/very high	26.1
**Any health risk factors**	**87.3**
*Socioeconomic disadvantage*	69.3
<10 years of education	48.3
Unemployed	44.6
Unstable accommodation	19.3
*Risky* s*ubstance use*	80.6
High risk/dependent alcohol consumption	50.1
Injecting drug use	52.4
>3 illicit drugs used	29.2

### Co-occurrence of morbidity

Table 2 shows the proportion of participants exhibiting morbidity across each pairwise combination of domains. Over half of the sample (58.8%) reported both risky substance use and socioeconomic disadvantage and more than two in five (44.3%) reported compromised physical and mental health. Among those who reported risky substance use, 60.0% also reported compromised physical health, and 47.7% also reported compromised mental health.

**Table 2:** **Co-occurrence of health-related morbidity across two domains**

	Poor physical health %	Poor mental health %	Socioeconomic disadvantage %
One or more markers of physical health			
One or more markers of poor mental health	44.3		
Socioeconomic disadvantage	49.6	39.6	
Risky substance use	60.0	47.7	58.8

Of the whole sample, 74.9% reported some form of health morbidity (i.e., poor physical and/or mental health) and risky substance use, and 69.7% reported overlapping health morbidity and socioeconomic disadvantage. Most reported co-occurring socio-economic disadvantage and risky substance use, as well as compromised mental health (94.1%) or physical health (96.9%).

Almost all participants (97.9%) reported morbidity in at least one health domain and 29.6% reported morbidity across all four domains: that is, almost a third of young adult prisoners exhibited an indicator of poor physical health, an indicator of poor mental health, an indicator of socioeconomic disadvantage and an indicator of risky substance use.

## Discussion

This study has offered important insights into a previously poorly explored area: overlapping health needs of young soon-to-be-released prisoners. Our findings suggest that most young prisoners experience compromised health across multiple domains, including physical and mental health, socioeconomic disadvantage and risky substance use. These overlapping health-related needs represent a significant public health concern, given that these soon-to-be released young prisoners are likely to experience a continuation or exacerbation of impairment as they transition out of prison (Kinner et al. 2011).

Although young people are one of Australia’s healthiest subgroups, they display age-specific morbidity patterns. In Australia, the majority of those aged under 25 years self-report ‘very good’ or ‘excellent’ health (AIHW Australian Institute of Health and Welfare 2011b). However, young people experience a higher burden of mental illness than adults, and report a higher prevalence of risky health-related behaviours, including tobacco, alcohol and other drug misuse, and risk-taking that leads to accidents and injury (Australian Institute of Health and Welfare 2007; Australian Institute of Health and Welfare 2011b). Poor health outcomes are a sensitive indicator of social and economic inequalities among this group: vulnerable young people have different health-related experiences than their less vulnerable peers and may require different forms of assistance from agencies. Vulnerability may also lead to greater involvement in risk-taking behaviours and risky situations, such as alcohol and drug misuse, unprotected sex and criminal behaviours (Gruskin et al. 2001).

Our findings suggest that young prisoners should be considered to be ‘vulnerable’. The vast majority of young prisoners in our sample were characterized by at least one health risk factor prior to their release from prison. Almost one in three experienced compromised physical *and* mental health, as well as socioeconomic disadvantage *and* risky substance use. The complexity of their presentations necessitates a high intensity, coordinated re-entry service to assist them in addressing multiple and complex issues as they transition out of prison.

Compared with the other indicators examined in this study relating to mental health, socioeconomic status and risky substance use, and with reports of older prisoners’ health (Australian Institute of Health and Welfare [Bibr CR5][Bibr CR43]; Fazel and Baillargeon 2011), young prisoners exhibited relatively good physical health, highlighting the long-term public health benefits of preventive interventions for this at-risk group, particularly in relation to STIs and chronic conditions. Physical wellbeing is an important asset for young prisoners transitioning into the community, because unlike some of their older peers (Australian Institute of Health and Welfare [Bibr CR5][Bibr CR43]; Fazel and Baillargeon 2011), most do not have illnesses or chronic conditions that limit their ability to address structural issues. For example, impaired mobility may limit ability to find new housing or restrict employment prospects (Van de Mheen et al. 1999). However, although young prisoners fare well compared with their older counterparts, it should be noted that compared with their peers in the community young prisoners appear to report poorer health, particularly in relation to HCV and STIs (Australian Institute of Health and Welfare 2011a). It is likely that compared with their peers who have not experienced incarceration, this group is less likely to access preventative health care and screening, has poorer health literacy and is unlikely to be targeted by community health promotion efforts. Further research is needed to determine how to better serve this group in relation to preventative health services, particularly in relation to infectious diseases.

Consistent with previous studies (Weinbaum et al. 2005; Van der Poorten et al. 2008), our findings indicate a high prevalence of HCV among young, soon-to-be-released ex-prisoners. In Australia, imprisonment is an independent risk factor for HCV transmission (Hellard et al. 2004; Dolan et al. 2010) and most HCV infections occur within a short time of initiating injecting (Maher et al. 2006), so young prisoners may benefit from both prevention and harm-reduction measures to reduce the spread of HCV within prison and in the community (Jurgens et al. 2009). Consistent with evidence that drug use is normative among the Australian prisoner population (Australian Institute of Health and Welfare 2010), many of the young prisoners in this study reported harmful alcohol use, polydrug use and/or injecting drug use prior to their incarceration. Substance misuse can increase the risk of poor short- and long-term health outcomes, particularly after release from custody, and the health- and justice-related consequences of IDU are severe (Merrell et al. 2010). Preventative interventions need to target young prisoners who have not commenced ‘careers’ of polydrug use, and particularly IDU.

### Overlapping morbidity complicates re-entry

Our findings highlight the complex health-related challenges that many young prisoners face when re-entering the community. In addition to addressing their criminal justice issues, they may have to contend with a multitude of co-occurring health and health-related needs. Previous research has highlighted that medical, psychiatric and substance use comorbidities complicate care and need to be simultaneously addressed (Altice et al. 2010). The concentration of health-related morbidity experienced by this group indicates a need for throughcare services that address overlapping health-related needs during the transition out of custody, and are accessible, acceptable and appropriate for young people. Young prisoners need to be assisted to address their overlapping health needs, including poor physical health (e.g., through HCV prevention programs), poor mental health (e.g., through low-threshold, youth-specific mental health services), social disadvantage (e.g., programs that assist young prisoners to access youth-specific accommodation, employment and social services), and risky substance use (e.g., through education and harm reduction programs around risky drug injecting behaviors).

### Study strengths and limitations

A key strength of this study is that by using Australia’s National Health Performance Framework to guide our data analysis, we have provided information on the health needs of young adult prisoners that is comparable to that used to guide community health system design in Australia. Through the use of this framework we have ensured that the health domains are consistent with those used with prisoners and the general population in Australia (Australian Institute of Health and Welfare 2010 Australian Institute of Health and Welfare 2011a).

However, our findings should be viewed in the context of several methodological issues. First, the focus of this study was to examine comorbidity at a macro level, to inform the coordination of service delivery. Given this health systems approach, we believe that such macro-level indicators are appropriate. Heterogeneity within our health-related domains is a limitation that we acknowledge, but it is an inherent limitation of a broad systems approach. Importantly from a policy perspective, our approach and indicators are consistent with that used to inform policy and service systems at a national level in Australia (Belcher and Al-Yaman 2007). We believe that a key strength of our approach lies in its direct comparability with this national system.

Similarly, to permit comparability with national (Australian) data we chose to use a lifetime (‘ever’) timeframe for our health indicators because this is the approach taken by the Australian Institute of Health and Welfare in its national prisoner health reports, and in its population-wide health collections (see for example Australian Institute of Health and Welfare 2010 and Australian Institute of Health and Welfare [Bibr CR5][Bibr CR43]). Another reason for choosing lifetime health indicators is that we collected data from prisoners at the end of their sentence, when they were most likely to have had their health issues addressed. Given evidence that historical health problems in prisoners often recur soon after release (Kinner 2006; Peterman et al. 2006) we believe that ‘current’ variables would under-ascertain health needs in this population.

Further, although we acknowledge that lifetime prevalence is not the same as point prevalence, for at least some of the health conditions we examine, past morbidity is a strong predictor of future morbidity. For example, past STIs are strong predictors of reinfection (Peterman et al. 2006) and lifetime diagnoses of other health issues are likely to be predictive of current impairments (Costello et al. 2002) since this population under-access health care and are unlikely to have received support to address the biopsychosocial issues associated with mental health and physical morbidity. Consequently, we are confident that our study paints a relatively accurate picture of the substantial overlapping health needs of young people leaving prison. However, health needs are likely to increase in the period immediately following release, particularly for those who experience homelessness and/or unemployment or return to pre-incarceration levels of substance use. Thus, our findings may be viewed as an underestimate of the overlapping health needs of young prisoners on return to the community.

The cross-sectional design precluded strong inferences about causal associations. Longitudinal studies investigating the health-related experiences of young people as they transition through prison and back to the community are required. Further, most health indicators were assessed by self-report; however, participants were likely to under-report rather than over-report drug-related behaviors (McGregor and Mikkai 2003), so it is unlikely that figures relating to risky substance use are over-estimates. An important feature of the health system in Australia is that unlike in the United States and some other countries, we have universal health insurance in the community. Although relying on self-reported health conditions has limitations, these limitations are less significant in the Australian context, given that all Australian prisons provide low-threshold, free healthcare for prisoners, and we sampled prisoners at the end of their sentence, and in doing so, aimed to maximise ascertainment of disease.

Finally, while our sample was reasonably representative of young, soon-to-be-released adult prisoners in Queensland, further research is required before the findings can be generalized to other jurisdictions in Australia or internationally.

### Policy implications

Our findings indicate a need for studies that evaluate the impact of youth-specific pilot programs aiming to reduce potential short- and long-term, health-related harms among young people leaving adult prison. In the UK, practitioners have focussed on improving youth transitions through the health-care system (Bolton-Maggs 2007; McDonagh 2007). The UK Department of Health’s (2006) best practice guideline ‘Transition: getting it right for young people’ mirrors the need for an evidence-based, best-practice guideline that can be applied in every Australian jurisdiction in relation to transitional practices for young prisoners. Support for prisoners should commence in prison and continue following release (Borzycki and Baldry 2003). However, there have been few evaluations of the health impacts of such programs and little is known about ‘what works’ to improve health-related outcomes among ex-prisoners in general. To inform policy about what constitutes ‘best practice', pilot studies of throughcare programs targeting young prisoners are needed.

National surveys of young people’s health should systematically report statistics for young prisoners, and emphasise that they are a group of ‘vulnerable’ young people who might need additional support and assistance from community services and agencies (Ahalt et al. 2012). In addition to transitioning out of prison, young ex-prisoners may be making transitions into adulthood (Arnett, 2001). For example, following release from prison, young prisoners may experience independent living or full-time employment for the first time. Therefore, to better understand young peoples’ transitions, data collection tools for future studies should include questions relating to the transition into young adulthood, and variables related to independent living, new relationships and first jobs.

In Australia, and other jurisdictions, separate health care systems in prisons and the community can complicate transition. This study demonstrates the need for integrated, collaborative approaches that move beyond ‘siloed’ health care and social service delivery. To date, prisoner health research has been characterised by a lack of data related to overlapping health needs, which can inform coordination of service delivery in prison and the community. This study has gone some way to addressing this issue, and has highlighted the need for further research that addresses the methodological gaps we have identified here.

## Conclusions

The majority of young prisoners will eventually return to the community, and their overlapping health-related needs represent a significant public health concern. To the authors’ knowledge, this is the first study internationally to present evidence about the overlapping health needs of young people soon to be released from adult prisons. By examining the prevalence and the co-occurrence of health needs among these young adult prisoners, our study has highlighted the need for proactive, appropriate, targeted throughcare coordinated across different health-related sectors.

## References

[CR1] Ahalt C, Binswanger I (2012). Confined to ignorance: the absence of prisoner information from nationally representative health data sets. J Gen Intern Med.

[CR2] Arnett JJ (2001). Conceptions of the transition to adulthood: perspectives from adolescence through midlife. J Adult Dev.

[CR3] Australian Institute of Health and Welfare (2007). Young Australians: Their Health and Wellbeing 2007. Key National Indicators.

[CR4] Australian Institute of Health and Welfare (2010). Australia’s Health 2010.

[CR5] Australian Institute of Health and Welfare (2010). The health of Australia's prisoners 2010.

[CR6] Australian Institute of Health and Welfare (2011). Young Australians: Their Health and Wellbeing 2011. Key National Indicators.

[CR7] Babor T, Higgins-Biddle J (2001). The Alcohol Use Disorders Identification Test: Guidelines for Use in Primary Care.

[CR8] Baldry E, McDonnell D (2004). The Role of Housing in Preventing Re-Offending.

[CR9] Belcher J, Al Yaman F (2007). Prisoner Health in Australia: Contemporary Information Collection and A Way Forward.

[CR10] Bird SM, Hutchinson SJ (2003). Male drugs-related deaths in the fortnight after release from prison: Scotland, 1996–99. Addiction.

[CR11] Bolton-Maggs P (2007). Transition of care from paediatric to adult services in haemotology. Arch Dis Child.

[CR12] Borzycki M, Baldry E (2003). Trends < issues in crime and criminal justice. Promoting Integration: The Provision of Post-release Services.

[CR13] Cauffmann E (2012). Aligning justice system processing with developmental science. Criminology < Public Policy.

[CR14] Costello EJ, Pine DS, Hammen C, March JS, Plotsky PM, Weissman MM, Biederman J, Goldsmith HH, Kaufman J, Lewinsohn PM, Hellander M, Hoagwood K, Koretz DS, Nelson CA, Leckman JF (2002). Development and natural history of mood disorders. Biol Psychiatry.

[CR15] Dolan K, Teutsch S (2010). Incidence and risk for acute hepatitis C infection during imprisonment in Australia. Eur J Epidemiol.

[CR16] Farrington DP, Loeber R, Howell JC (2012). Young adult offenders. Criminology < Public Policy.

[CR17] Fazel S, Baillargeon J (2011). The health of prisoners. Lancet.

[CR18] Fazel S, Bains P (2006). Substance abuse and dependence in prisoners: a systematic review. Addiction.

[CR19] Friedmann P, Green T (2012). Collaborative behavioral management among parolees: drug use, crime and re-arrest in the step’n out randomized trial. Addiction.

[CR20] Gendreau P, Little T (1996). A meta-analysis of the predictors of adult offender recidivism: what works!. Criminology.

[CR21] Gruskin S, Plafker K (2001). Understanding and responding to youth substance use: the contribution of a health and human rights framework. Am J Public Health.

[CR22] Hammett TM, Roberts C (2001). Health-related issues in prisoner reentry. Crime Delinquency.

[CR23] Hellard M, Hocking J (2004). The prevalence and the risk behaviours associated with the transmission of hepatitis C virus in Australian correctional facilities. Epidemiol Infect.

[CR24] Holland S, Pointon K (2007). Corrections Research Paper Series C. Who Returns To Prison? Patterns of Recidivism Among Prisoners Released From Custody in Victoria in 2002–03.

[CR25] Jurgens R, Ball A (2009). Interventions to reduce HIV transmission related to injecting drug use in prison. Lancet Infect Dis.

[CR26] Kariminia A, Law MG (2007). Suicide risk among recently released prisoners in New South Wales, Australia. MJA.

[CR27] Kessler RC, Barker PR, Colpe LJ, Epstein JF, Gfroerer JC, Hiripi E, Howes MJ, Normand SL, Manderscheid RW, Walters EE, Zaslavsky AM (2003). Screening for serious mental illness in the general population. Arch Gen Psychiatry.

[CR28] Kessler RD, Berglund P (2005). Lifetime prevalence and age-of-onset distributions of *DSM-IV* disorders in the national comorbidity survey replication. Arch Gen Psychiatry.

[CR29] Kinner S (2006). Continuity of health impairment and substance misuse among adult prisoners in Queensland, Australia. Int J Prison Health.

[CR30] Kinner S (2008). Passports to advantage: health and capacity building as a basis for social integration. Flinders Journal of Law Reform.

[CR31] Kinner S, Cogger S (2007). The Post-Release Experiences of Prisoners in Queensland: Drug use, crime and social integration (NDSLEFC Report).

[CR32] Kinner S, Lennox N (2009). Randomised controlled trial of a post-release intervention for prisoners with and without an intellectual disability. Journal on Developmental Disabilities.

[CR33] Kinner S, Preen D (2011). Counting the cost: estimating the number of deaths among recently release prisoners in Australia. MJA.

[CR34] Kinner S, Streitberg L, Butler T, Levy M (2012). Prisoner and ex-prisoner health: improving access to primary care. Aust Fam Physician.

[CR35] Lattimore P, Visher C (2009). The Multi-Site Evaluation of SVORI: Summary and Synthesis, National Institute of Justice.

[CR36] Maher L, Jalaludin B (2006). Incidence and risk factors for hepatitis C seroconversion in injecting drug users in Australia. Addiction.

[CR37] Mallik-Kane K, Visher CA (2008). Health and Prisoner Reentry: How Physical, Mental and Substance Abuse Conditions Shape the Process of Reintegration.

[CR38] McDonagh J (2007). Transition of care from paediatric to adult rheumatology. Arch Dis Child.

[CR39] McGregor K, Mikkai T (2003). Trends and Issues. Self-Reported Drug Use: How Prevalent Is Under-Reporting?.

[CR40] Merrell ELC, Kariminia A (2010). Meta-analysis of drug-related deaths soon after release from prison. Addiction.

[CR41] Saunders J, Aasland O (1993). Development of the alcohol use disorders identifications test (AUDIT): WHO collaborative project on early detection of persons with harmful alcohol consumption - II. Addiction.

[CR42] StataCorp (2009). Stata Statistical Software: Release 11.

[CR43] Peterman, TA, Tian, LH, Metcalf, CA, Satterwhite, CL, Malotte, KC, DeAugustine, N, Paul, SM, Cross, H, Rietmeijer, CA, Douglas, JM Jr, RESPECT-2 Study Group (2006). High incidence of new sexually transmitted infections in the year following a sexually transmitted infection: a case for rescreening. Ann Intern Med.

[CR44] Australian Institute of Health and Welfare (2011). Cat. no. PHE 149. The Health of Australia’s Prisoners 2010.

[CR45] Van de Mheen H, Strongs K (1999). The influence of adult ill health on occupational class mobility and mobility out of and into employment in The Netherlands. Soc Sci Med.

[CR46] Van der Poorten D, Kenny DT (2008). Prevalence of and risk factors for hepatitis C in Aboriginal and non-Aboriginal adolescent offenders. Med J Aust.

[CR47] Van Dooren K, Claudio F, Kinner SA, Williams M (2011). Beyond reintegration: a framework for understanding ex-prisoner health. Int J Prison Health.

[CR48] van Dooren K, Forsyth S, Kinner SA: Risk of death for young ex-prisoners in the year following release from adult prison. *Aust N Z J Public Health* in press. 10.1111/1753-6405.12087 23895482

[CR49] Weinbaum C, Sabin K (2005). Hepatitis B, hepatitis C, and HIV in correctional populations: a review of epidemiology and prevention. AIDS.

[CR50] Wilson J, Zozula C (2012). Risk, recidivism, and (re)habilitation. Prison J.

[CR51] Woolard JL (2012). Young adults. Criminology < Public Policy.

